# Imaging-AMARETTO: An Imaging Genomics Software Tool to Interrogate Multiomics Networks for Relevance to Radiography and Histopathology Imaging Biomarkers of Clinical Outcomes

**DOI:** 10.1200/CCI.19.00125

**Published:** 2020-05-08

**Authors:** Olivier Gevaert, Mohsen Nabian, Shaimaa Bakr, Celine Everaert, Jayendra Shinde, Artur Manukyan, Ted Liefeld, Thorin Tabor, Jishu Xu, Joachim Lupberger, Brian J. Haas, Thomas F. Baumert, Mikel Hernaez, Michael Reich, Francisco J. Quintana, Erik J. Uhlmann, Anna M. Krichevsky, Jill P. Mesirov, Vincent Carey, Nathalie Pochet

**Affiliations:** ^1^ Stanford Center for Biomedical Informatics Research, Department of Medicine and Biomedical Data Science, Stanford University, Stanford, CA; ^2^ Cell Circuits Program, Broad Institute of MIT and Harvard, Cambridge, MA; ^3^ Ann Romney Center for Neurologic Diseases, Department of Neurology, Brigham and Women’s Hospital, Harvard Medical School, Boston, MA; ^4^ Department of Medicine, University of California, San Diego, San Diego, CA; ^5^ Rush University Medical Center, Chicago, IL; ^6^ INSERM, U1110, Institut de Recherche sur les Maladies Virales et Hépatiques, Université de Strasbourg, Institut Hopitalo-Universitaire, Hôpitaux Universitaires de Strasbourg, Strasbourg, France; ^7^ Carl R. Woese Institute for Genomic Biology, University of Illinois at Urbana-Champaign, Champaign, IL; ^8^ Channing Division of Network Medicine, Brigham and Women’s Hospital, Harvard Medical School, Boston, MA

## Abstract

**PURPOSE:**

The availability of increasing volumes of multiomics, imaging, and clinical data in complex diseases such as cancer opens opportunities for the formulation and development of computational imaging genomics methods that can link multiomics, imaging, and clinical data.

**METHODS:**

Here, we present the Imaging-AMARETTO algorithms and software tools to systematically interrogate regulatory networks derived from multiomics data within and across related patient studies for their relevance to radiography and histopathology imaging features predicting clinical outcomes.

**RESULTS:**

To demonstrate its utility, we applied Imaging-AMARETTO to integrate three patient studies of brain tumors, specifically, multiomics with radiography imaging data from The Cancer Genome Atlas (TCGA) glioblastoma multiforme (GBM) and low-grade glioma (LGG) cohorts and transcriptomics with histopathology imaging data from the Ivy Glioblastoma Atlas Project (IvyGAP) GBM cohort. Our results show that Imaging-AMARETTO recapitulates known key drivers of tumor-associated microglia and macrophage mechanisms, mediated by *STAT3*, *AHR*, and *CCR2*, and neurodevelopmental and stemness mechanisms, mediated by *OLIG2*. Imaging-AMARETTO provides interpretation of their underlying molecular mechanisms in light of imaging biomarkers of clinical outcomes and uncovers novel master drivers, *THBS1* and *MAP2*, that establish relationships across these distinct mechanisms.

**CONCLUSION:**

Our network-based imaging genomics tools serve as hypothesis generators that facilitate the interrogation of known and uncovering of novel hypotheses for follow-up with experimental validation studies. We anticipate that our Imaging-AMARETTO imaging genomics tools will be useful to the community of biomedical researchers for applications to similar studies of cancer and other complex diseases with available multiomics, imaging, and clinical data.

## INTRODUCTION

Major collaborative initiatives have unleashed a myriad of multiomics, clinical, and imaging data for large patient cohorts in studies of cancer, such as multiomics and clinical data from The Cancer Genome Atlas (TCGA)^1^ and the Clinical Proteomic Tumor Analysis Consortium (CPTAC)^2^ and radiographic and histopathology imaging data from The Cancer Imaging Archive (TCIA).^3^ For example, the brain tumor section of TCGA provides multiomics profiles, including RNA sequencing (RNA-seq), DNA copy number variation, and DNA methylation data for approximately 500 patients with glioblastoma multiforme (GBM)^4,5^ and approximately 500 patients with low-grade glioma (LGG).^6^ The TCIA Visually AcceSAble Rembrandt Images (VASARI)^7-9^ project curated a feature set of approximately 30 magnetic resonance imaging (MRI)–derived features on the basis of specialists’ review that is available for approximately 200 patients with GBM and approximately 180 patients with LGG.

**CONTEXT**

**Key Objective**
Imaging-AMARETTO provides software tools for imaging genomics through multiomics, clinical, and imaging data fusion within and across multiple patient studies of cancer, toward better diagnostic and prognostic models of cancer.
**Knowledge Generated**
Our network-based imaging genomics tools serve as powerful hypothesis generators that facilitate the testing of known hypotheses and uncovering of novel hypotheses for follow-up with experimental validation studies. Our case study that integrated multiple studies of brain cancer illustrates how Imaging-AMARETTO can be used for imaging diagnostics and prognostics by interrogating multimodal and multiscale networks for imaging biomarkers to identify their clinically relevant underlying molecular mechanisms.
**Relevance**
We anticipate that our Imaging-AMARETTO tools for network-based fusion of multiomics, clinical, and imaging data will directly lead to better diagnostic and prognostic models of cancer. In addition, our tools for network biology and medicine will open new avenues for drug discovery by integrating pharmacogenomics data into these networks, toward better therapeutics of cancer.

A trade-off exists between the number of patients included in a data set and the depth of analysis that has moved to increasing levels of refinement, ranging from studying tissues, to cell populations, to single-cell^10-12^ sequencing. For example, the Ivy Glioblastoma Atlas Project (IvyGAP)^13-15^ provides 270 transcriptomic profiles refined through histopathology imaging and annotated by a consensus of histopathologists for studying anatomic structures and cancer stem cells for a subset of approximately 30 patients from the TCGA GBM cohort.

In parallel, quantitative imaging provides tools that are capable of processing large volumes of radiography and histopathology images, such as deep convolutional neural networks. The promising field of radiogenomics is based on the idea that entities at different scales, such as molecules, cells, and tissues, are linked to one another and, therefore, may be modeled as a whole.^16^ Studies have shown that quantitative image features extracted from radiography imaging data are associated and predictive of gene expression patterns from tissues of matched tumors.^17-20^ Recent efforts further expand this work to link multiomics data with both radiography and histopathology imaging,^21,22^ toward developing methods for imaging genomics.

These large archives of multimodal and multiscale data sources provide complementary insights into the mechanistic basis of cancer, toward better diagnosis and treatment, and open unprecedented modeling opportunities to link multiomics data with clinical and imaging phenotypes. As a solution to imaging genomics, we introduce the Imaging-AMARETTO software tools to systematically interrogate networks derived from multiomics data for relevance to imaging biomarkers of clinical outcomes. We demonstrate the utility of these imaging genomics tools by integrating three patient brain tumor databases, including the TCGA GBM and LGG and the IvyGAP GBM cohorts. We uncover known and novel drivers of tumor-associated microglia and macrophage mechanisms, and neurodevelopmental and stemness mechanisms, with interpretation of the underlying molecular mechanisms in light of imaging biomarkers of clinical outcomes.

## METHODS

### The *AMARETTO Software Architecture

**FIG 1.**
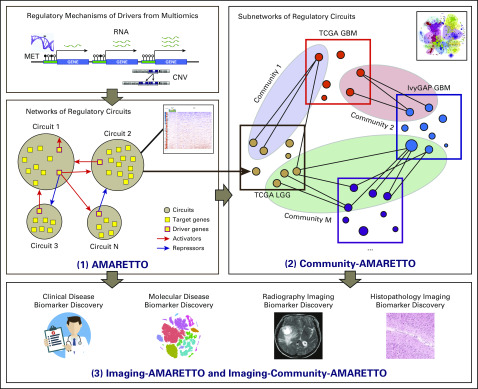
The Imaging-AMARETTO and Imaging-Community-AMARETTO software architecture. The overall framework offers modular and complementary solutions to multimodal and multiscale aspects of network-based modeling within and across multiple studies of cancer. Specifically, (1) The AMARETTO algorithm learns networks of regulatory modules or circuits (circuits of drivers and target genes) from functional genomics or multiomics data (eg, DNA copy number variation [CNV], DNA methylation [MET], RNA gene expression [RNA]) within each study of cancer (eg, within The Cancer Genome Atlas [TCGA] glioblastoma multiforme [GBM], Ivy Glioblastoma Atlas Project [IvyGAP] GBM, or TCGA low-grade glioma [LGG] cohorts separately); (2) the Community-AMARETTO algorithm learns communities or subnetworks of regulatory circuits that are shared or distinct across networks derived from multiple studies of cancer (eg, across the TCGA GBM, IvyGAP GBM, and TCGA LGG cohorts); and (3) the Imaging-AMARETTO and Imaging-Community-AMARETTO algorithms associate these circuits (AMARETTO) and subnetworks (Community-AMARETTO) to clinical, molecular, and imaging-derived biomarkers by mapping radiography and histopathology imaging data onto the networks and assessing their clinical relevance for imaging diagnostics.

The *AMARETTO framework (Fig 1) provides tools for network-based fusion of multiomics, clinical, and imaging data within and across multiple patient studies of cancer. Specifically, this framework offers modular and complementary solutions to multimodal and multiscale aspects of network-based modeling within and across studies of cancer through the AMARETTO and Community-AMARETTO algorithms, respectively. In this work, we present an imaging genomics software toolbox that comprises the newly formulated Imaging-AMARETTO and Imaging-Community-AMARETTO algorithms that together facilitate interpretation of patient-derived multiomics networks for their relevance to radiography and histopathology imaging biomarkers of clinical outcomes. Resources of the *AMARETTO^23^ software toolbox are available through GitHub, Bioconductor, R Jupyter Notebook, GenePattern, GenomeSpace, and GenePattern Notebook.

### Imaging-AMARETTO From a User’s Perspective

The workflow for multiomics, clinical, and imaging data fusion includes utilities to learn networks from individual patient cohorts using Imaging-AMARETTO and to link networks across multiple related patient cohorts using Imaging-Community-AMARETTO. Together, these workflows allow users to integrate patient tumor-derived multiomics or transcriptional profiles with clinical and molecular characteristics and radiography and histopathology imaging features within and across related patient cohorts. The Imaging-AMARETTO source code in R is available from GitHub.^24^ An R Jupyter Notebook that provides stepwise guidelines for running the source code directly from GitHub for its application to brain cancer is available from Google Colaboratory.^25^

Imaging-AMARETTO supports multiple workflows: a patient cohort with only transcriptional profiles and a patient cohort with multiomics profiles. When only RNA-seq data are available for a cohort, a predefined list of candidate driver genes is required, which can be selected or uploaded by the user. Predefined lists of known drivers are available for collections of transcription factors (TFutils^26,27^ and Molecular Signatures Database [MSigDB]^28,29^ C3) and cancer driver genes (COSMIC^30^ Cancer Gene Census, MSigDB Hallmark). When genetic or epigenetic data are also available, they can help to guide the selection of candidate driver genes. Potential cancer drivers are identified as somatic recurrent cancer aberrations from genetic and epigenetic data sources using GISTIC^31^ and MethylMix.^32,33^ The GISTIC algorithm is used to identify copy number amplifications and deletions from DNA copy number variation data. The MethylMix algorithm is used to identify hyper- and hypomethylated sites from DNA methylation data. The user also specifies the number of regulatory modules to be learned from the data and the percentage of most varying genes to be included in the analysis. Networks of regulatory modules are inferred from RNA-seq data using an iterative optimization procedure. The algorithm^34-37^ starts with an initialization step that clusters the genes into modules of co-expressed target genes. For each of these modules, we learn the regulatory programs as a linear combination of candidate driver genes that best predict their target genes’ expression using Elastic Net–regularized regression.^38^ Target genes are then reassigned to the regulatory programs that best explain their gene expression levels as estimated by the predictive power of the regulatory programs’ respective regularized regression models when predicting the target genes’ expression.^34-37^ The algorithm iterates over these two steps until convergence. This analysis generates a network of regulatory modules, defined as a group of target genes collectively activated or repressed by their associated drivers.

### Imaging-Community-AMARETTO: Linking Imaging-AMARETTO Networks Across Cohorts

To compare and integrate networks of regulatory modules across multiple cohorts, the user can submit two or more Imaging-AMARETTO networks and optionally add known networks as collections of signatures to guide subnetwork learning and interpretation, such as immune cell (CIBERSORT^39,40^) and stemness^41-43^ signatures. The algorithm^34^ creates a module map of all pairwise comparisons between modules across multiple networks to assess the extent of overlapping genes between all pairs of modules (−log_10_ *P* value, hypergeometric test). This module map is partitioned using an edge betweenness community detection algorithm (Girvan-Newman^44^) that groups the modules into subnetworks or communities across the multiple networks. These communities represent shared behavior across two or more cohorts, and modules not assigned to communities are reported as distinct behavior specific for each cohort. This analysis generates subnetworks or communities of regulatory modules that are shared or distinct across multiple Imaging-AMARETTO networks derived from multiple cohorts and further refines shared and distinct behavior of modules with respect to their specific drivers.

### Downstream Utility for Interpreting Clinical and Experimental Outcomes

We developed several downstream utilities that facilitate interpretation of the Imaging-AMARETTO networks, including functional characterization, driver validation, clinical correlation, and imaging association. To functionally characterize modules and communities, we provide signatures from known gene sets databases (MSigDB) that can be augmented with user-defined signatures, such as immune cell,^39,40^ stromal cell,^45^ and stemness^41-43^ signatures. Regulatory modules and communities are assessed for enrichment in these known functional categories (hypergeometric test).

To validate the predicted drivers as regulators of their targets in modules and communities, we assess whether activator or repressor drivers have a direct or indirect impact on their targets using experimental genetic perturbation data. The user can test signatures derived from genetic perturbation experiments, such as signatures of target genes bound to transcription factors measured in protein-DNA–binding chromatin immunoprecipitation sequencing (ChIP-Seq) experiments (Encyclopedia of DNA Elements,^46^ ChIP-X Enrichment Analysis,^47^ Harmonizome^48^) or defined by motif binding (MSigDB C3), or signatures of genes induced or repressed in response to genetic knockdown or overexpression experiments of drivers (Library of Integrated Network-Based Cellular Signatures [LINCS]/Connectivity Map [CMAP],^49,50^ Harmonizome).

To characterize regulatory modules for clinical outcomes and molecular biomarkers, the user can submit phenotypes known for all or subsets of samples and specify the statistical hypothesis tests to use for each phenotype. Examples of clinical and molecular phenotypes include survival data, molecular subclasses (eg, mesenchymal, proneural, or classical GBM,^51^ astrocytoma or oligodendroglioma LGG) and biomarkers (eg, *IDH* mutation, *EGFR* amplification, *MGMT* methylation status). Our implementation supports survival analysis using Cox proportional hazards regression, nominal two-class and multiclass analysis using the Wilcoxon rank sum and Kruskal-Wallis tests, and continuous or ordinal analysis using the Pearson linear and Spearman rank correlation tests. These clinical and molecular phenotype associations are assessed for each of the regulatory modules in individual cohorts and combined for communities across cohorts.

Finally, to interpret regulatory modules for relevance to radiography or histopathology imaging features, associations with these imaging features can be assessed. Examples of radiography and histopathology phenotypes include the 30 TCIA VASARI MRI features defined by expert consensus for the TCGA GBM and LGG cohorts, the IvyGAP^13-15^ histopathology imaging features characterizing RNA-seq samples refined for anatomic structures and cancer stem cells defined by expert consensus for the IvyGAP GBM cohort, and radiography and histopathology imaging features derived using quantitative imaging methods.^20,52-54^

**FIG 2.**
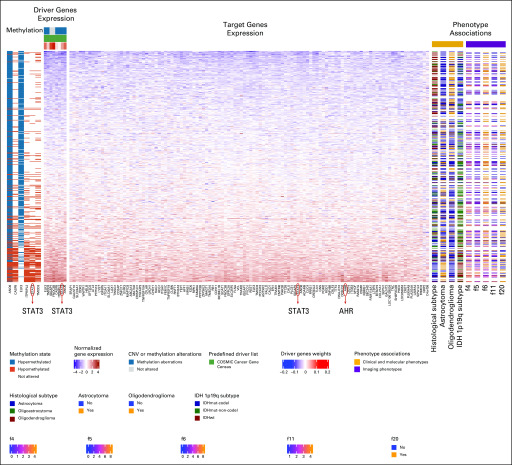
Imaging-AMARETTO predicts *STAT3* and *AHR* as known drivers of tumor-associated microglia and macrophage mechanisms in low-grade glioma (LGG). These heat maps present module 125 from The Cancer Genome Atlas (TCGA) LGG cohort that is a member of community 5 shared across the three cohorts (TCGA glioblastoma multiforme [GBM], Ivy Glioblastoma Atlas Project GBM, and TCGA LGG). For all patient-derived samples (rows), the heat maps show driver genes’ multiomics profiles (columns), including DNA methylation and RNA gene expression data (left panels); target genes’ (columns) RNA gene expression levels (middle panel); and relevant biomarkers (columns), including clinical and molecular and imaging phenotypes (right panels). This module includes eight driver genes (*FNDC3B, IQGAP1, ANO6, ELK3, STAT3, TMOD3, CASP8*, and *ITPRIPL2*) that jointly act as activator drivers of the 114 target genes in this module, including *AHR* and *STAT3*. Six driver genes are methylation driven (hypomethylation of *ANO6, CASP8, ITPRIPL2, STAT3*, and *TMOD3* and hypermethylation of *ANO6* and *ELK3*, inversely associated with their gene expression levels). Survival analysis reveals that increased expression of the genes in this module is associated with shorter survival (*P* = 7.3e-11; false discovery rate [FDR] = 1.0e-9). This module distinguishes between the histological subtypes (*P* = 1.6e-15; FDR = 2.1e-14), with lower expression representing the oligodendroglioma subtype (*P* = 1.0e-14; FDR = 1.1e-13) and higher expression reflecting the astrocytoma subtype (*P* = 1.5e-11; FDR = 2.8e-10). *IDH* mutation (mut) status and 1p19q subtypes are associated with the module expression (*P* = 3.9e-22; FDR = 1.5e-21), with higher expression levels representing the wild-type (wt) status. Association of Visually AcceSAble Rembrandt Images magnetic resonance imaging features with this module shows that the proportion of enhancing tumor (f5; *P* = 8.96e-7; FDR = 0.0000134) and enhancement intensity (f4; *P* = .0000395; FDR = 0.000494) are correlated with gene expression, while the proportions of nonenhancing tumor (f6; *P* = .0000452; FDR = 0.00036) and cortical involvement (f20; *P* = .0175; FDR = 0.118) are inversely correlated with expression. Module gene expression levels also distinguish between the thicknesses of enhancing margin (f11; *P* = .00137; FDR = 0.02).^71^ CNV, copy number variation.

**FIG 3.**
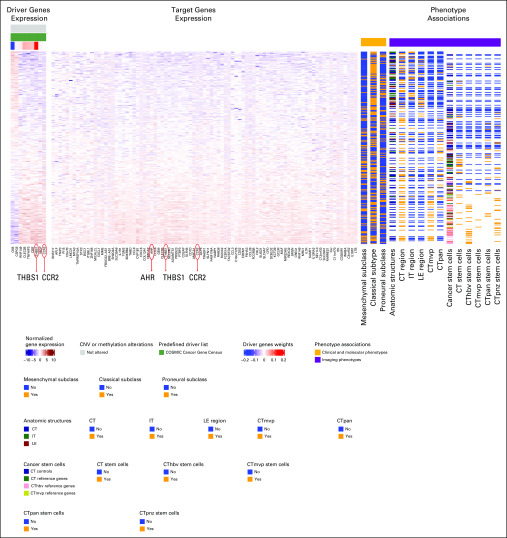
Imaging-AMARETTO predicts *AHR* and *CCR2* as known drivers and identifies *TBHS1* as a novel driver of tumor-associated microglia and macrophage mechanisms in glioblastoma multiforme (GBM). These heat maps present module 64 from the Ivy Glioblastoma Atlas Project (IvyGAP) GBM cohort that is a member of community 5 shared across the three cohorts (The Cancer Genome Atlas [TCGA] GBM, IvyGAP GBM, and TCGA low-grade glioma). For all patient-derived samples (rows), the heat maps show driver genes’ functional genomics profiles (columns), specifically RNA gene expression profiles (left panel); target genes’ (columns) RNA gene expression levels (middle panel); and relevant biomarkers (columns), including clinical and molecular and imaging phenotypes (right panels). This module includes nine driver genes (*THBS1, CLEC2B, TNFAIP3, DSE, RNF149, MGP, CCR2, CSPG5*, and *CKB*) that jointly act as activators (eg, *CCR2*, a squamous cell carcinoma tumor-rejection antigen recognized by T lymphocytes and candidate for specific immunotherapy) and repressors (*CSPG5* and *CKB*) that drive the 87 target genes in this module, including *AHR, CCR2*, and *THBS1*. Association analyses confirm that higher expression of module genes reflects samples derived from patients in the mesenchymal subclass (*P* = .000047; false discovery rate [FDR] = 0.00015), while lower expression represents the classical subclass (*P* = .032; FDR = 0.067). Association of histopathology imaging features that study anatomic structures reveals that genes in this module distinguish between the samples derived from distinct anatomic structures (*P* = 1.8e-15; FDR = 4.3e-15), (continued on following page)where cellular tumor (CT; *P* = .0000078; FDR = 0.000013) samples and, in particular, with substructure microvascular proliferation (CTmvp; *P* = 8.7e-14; FDR = 6.2e-13) and pseudopalisading cells around necrosis (CTpan; *P* = .018; FDR = 0.027) show elevated expression of module genes, while leading edge (LE; *P* = .018; FDR = 0.023) and infiltrating tumor (IT; *P* = .0014; FDR = 0.0045) samples show lower expression. Association of histopathology imaging features targeting cancer stem cells reveals that samples derived from cancer stem cells are generally associated with higher module gene expression compared with nonstem cells (*P* = 4.0e-16; FDR = 8.6e-15) and, specifically, elevated expression in stem-cell *v* control samples from substructures of the CT (*P* = .0000059; FDR = 0.00018), including hyperplastic blood vessels (CThbv; *P* = 1.2e-11; FDR = 5.2e-10), perinecrotic zone (CTpnz; *P* = 3.4e-9; FDR = 3.4e-8), CTpan (*P* = .000087; FDR = 0.00032), and CTmvp (*P* = .0039; FDR = 0.029).^72^ CNV, copy number variation.

**FIG 4.**
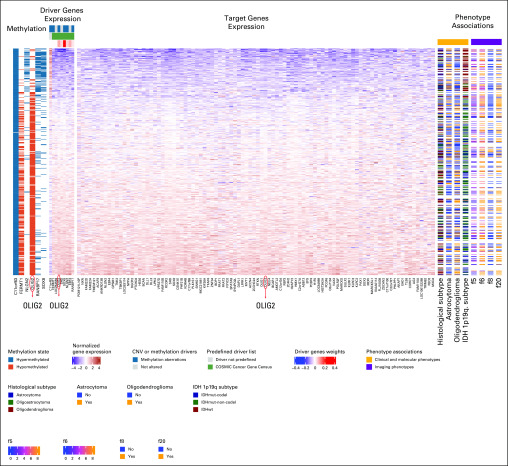
Imaging-AMARETTO predicts *OLIG2* as known driver of neurodevelopmental and stemness mechanisms in low-grade glioma (LGG). These heat maps present module 91 from The Cancer Genome Atlas (TCGA) LGG cohort that is a member of community 2 shared across the three cohorts (TCGA glioblastoma multiforme [GBM], Ivy Glioblastoma Atlas Project GBM, and TCGA LGG). For all patient-derived samples (rows), the heat maps show driver genes’ multiomics profiles (columns), including DNA methylation and RNA gene expression data (left panels); target genes’ (columns) RNA gene expression levels (middle panel); and relevant biomarkers (columns), including clinical and molecular and imaging phenotypes (right panels). This module includes nine driver genes (*SOX8, OLIG2, EBF4, NLGN2, SHD, RANBP17, FERMT1, LOC254559*, and *C11orf63*) that jointly act as activator and repressor (*C11orf63*) drivers of the 94 target genes in this module, including *OLIG2*. Six driver genes are methylation driven (hypomethylation of *FERMT1* and *OLIG2* and hypermethylation of *C11orf63, NLGN2, RANBP17*, and *SOX8*, inversely associated with their gene expression levels). Survival analysis reveals that increased expression of the genes in this module is associated with better survival (*P* = 7.1e-11; false discovery rate [FDR] = 1.0e-9). This module distinguishes between the histological subtypes (*P* = .00047; FDR = 0.00084), with lower expression representing the astrocytoma subtype (*P* = .00016; FDR = 0.00039) and higher expression reflecting the oligodendroglioma subtype (*P* = .0027; FDR = 0.0047). *IDH* mutation (mut) status and 1p19q subtypes are associated with the module expression (*P* = 2.0e-26; FDR = 1.1e-25), with lower expression levels representing the wild-type (wt) status. Association of Visually AcceSAble Rembrandt Images magnetic resonance imaging features with this module shows that the proportion of enhancing tumor (f5; *P* = .016; FDR = 0.032) is inversely correlated with gene expression, while the proportion of nonenhancing tumor (f6; *P* = .032; FDR = 0.066), cortical involvement (f20; *P* = .018; FDR = 0.12), and the presence of cysts (f8; *P* = .0095; FDR = 0.14) are correlated with expression.^73^ CNV, copy number variation.

**FIG 5.**
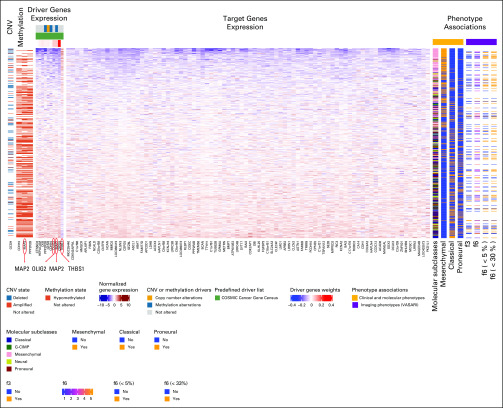
Imaging-AMARETTO predicts *OLIG2* as known driver and identifies *MAP2* and *THBS1* as novel drivers of neurodevelopmental and stemness mechanisms in glioblastoma multiforme (GBM). These heat maps present module 75 from The Cancer Genome Atlas (TCGA) GBM cohort that is a member of community 2 shared across the three cohorts (TCGA GBM, Ivy Glioblastoma Atlas Project GBM, and TCGA low-grade glioma). For all patient-derived samples (rows), the heat maps show driver genes’ multiomics profiles (columns), including DNA copy number variation (CNV), DNA methylation, and RNA genes’ expression data (left panels); target genes’ (columns) RNA gene expression levels (middle panel); and relevant biomarkers (columns), including clinical and molecular and imaging phenotypes (right panels). This module includes 10 driver genes (*CSPG5, MAP2, OLIG2, CKB, GCSH, PPP2R2B, HEPACAM, CDH10, CTNND2*, and *THBS1*) that jointly act as activator and repressor (*THBS1*) drivers of the 84 target genes in this module. One driver gene, *GCSH*, is copy number driven (somatic recurrent copy number deletions and amplifications associated with its gene expression), and three driver genes are methylation driven (hypomethylation of *CDH10*, *MAP2*, and *PPP2R2B*, inversely associated with their gene expression). Higher expression of genes in this module comprises the classical (*P* = 9.9e-13; false discovery rate [FDR] = 5.1e-12) and proneural (*P* = .0000024; FDR = 0.0000056) molecular subclasses, while lower expression of genes in this module represents the mesenchymal molecular subclass (*P* = 6.1e-40; FDR = 2.3e-38). Association of Visually AcceSAble Rembrandt Images (VASARI) magnetic resonance imaging features with this module shows that the proportion of nonenhancing tumor (f6; *P* = .013; FDR = 0.060, with nonenhancing proportion < 33%, *P* = .0014; FDR = 0.027, and nonenhancing proportion < 5%, *P* = .032; FDR = 0.17) and speech receptive eloquent cortex (f3; *P* = .046; FDR = 0.75) are correlated with the module gene expression.^74^

**FIG 6.**
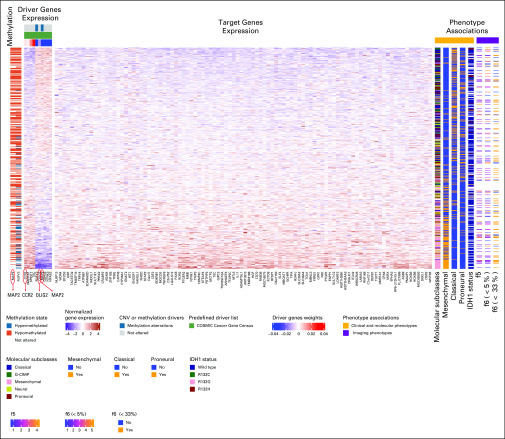
Imaging-AMARETTO predicts *CCR2* and *OLIG2* as co-acting known activator and repressor drivers and *MAP2* as novel repressor driver linking tumor-associated microglia and macrophage mechanisms with neurodevelopmental and stemness mechanisms in glioblastoma multiforme (GBM). These heat maps present module 98 from The Cancer Genome Atlas (TCGA) GBM cohort that is a member of community 2 shared across the three cohorts (TCGA GBM, Ivy Glioblastoma Atlas Project GBM, and TCGA low-grade glioma). For all patient-derived samples (rows), the heat maps show driver genes’ multiomics profiles (columns), including DNA methylation and RNA gene expression data (left panels); target genes’ (columns) RNA gene expression levels (middle panel); and relevant biomarkers (columns), including clinical and molecular and imaging phenotypes (right panels). This module includes 10 driver genes (*TEC, SPINT1, CCR2, MAN1A1, OLIG2, NPAS3, MAP2, RUFY3, NTRK3*, and *CSPG5*) that jointly act as activator (*TEC, SPINT1, CCR2, MAN1A1*) and repressor (*OLIG2, NPAS3, MAP2, RUFY3, NTRK3, CSPG5*) drivers of the 98 target genes in this module. Two driver genes, *MAP2* and *RUFY3*, are methylation driven (hypomethylation of *MAP2* and hyper- and hypomethylation of *RUFY3,* inversely associated with their gene expression levels). Higher expression of the genes in this module represents the mesenchymal molecular subclass (*P* = 5.1e-35; false discovery rate [FDR] = 5.9e-34), while lower expression of genes in this module comprises the classical (*P* = 6.1e-15; FDR = 3.8e-14), G-CIMP (*P* = .000012; FDR = 0.000030), and proneural (*P* = .00034; FDR = 0.00062) molecular subclasses. *IDH1* mutation status is associated with the module expression (*P* = .00069; FDR = 0.0019), with higher expression levels representing the wild-type status. Association of Visually AcceSAble Rembrandt Images magnetic resonance imaging features with this module shows that the proportion of nonenhancing tumor is inversely correlated (f6; *P* = .045; FDR = 0.13) with nonenhancing proportion < 33% (*P* = .0086; FDR = 0.067) and the proportion of enhancing tumor is correlated (f5; *P* = .020; FDR = 0.28) with the module gene expression.^75^

**FIG A1.**
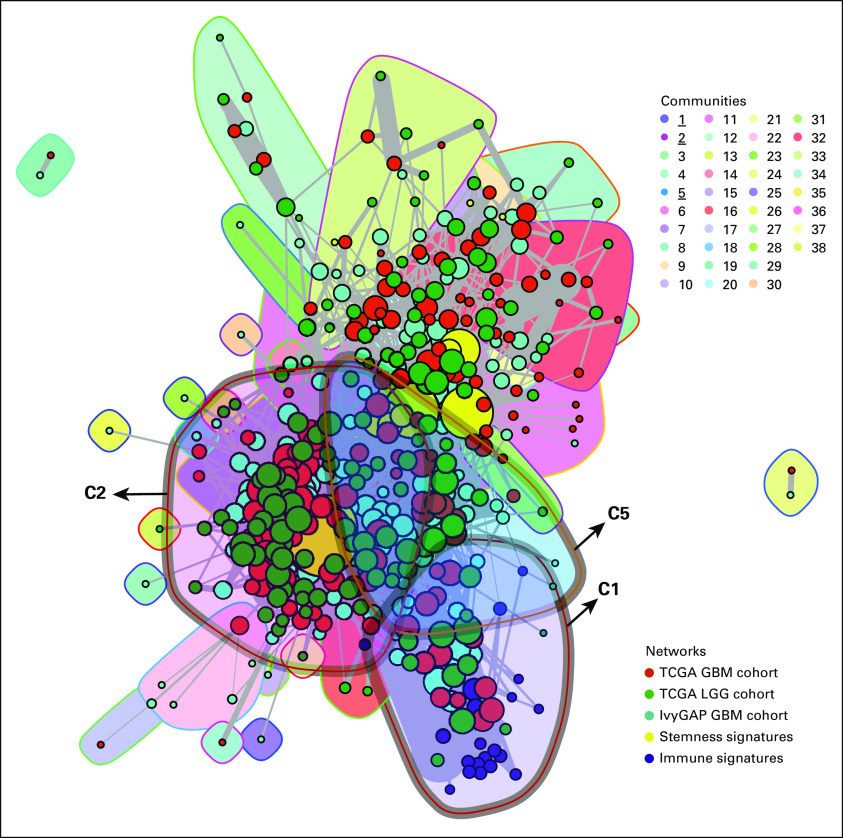
Imaging-Community-AMARETTO identifies known drivers *AHR, STAT3, CCR2*, and *OLIG2* and uncovers novel master drivers *THBS1* and *MAP2* that link distinct key mechanisms that underlie glioma. In this Imaging-Community-AMARETTO graph, the nodes represent the regulatory modules or circuits that are learned from the three studies of brain cancer (The Cancer Genome Atlas [TCGA] glioblastoma multiforme [GBM], Ivy Glioblastoma Project [IvyGAP] GBM, and TCGA low-grade glioma [LGG]; node sizes are scaled by the number of driver and target genes in the modules), the edges represent the extent of overlapping genes between the modules across the three cohorts (edge thickness is scaled with the significance of the overlapping genes between modules), and the clouds represent how the modules across the three cohorts are grouped into the communities or subnetworks that are learned using the Girvan-Newman edge betweenness community detection algorithm. Imaging-Community-AMARETTO organized modules regulated by known drivers of tumor-associated microglia and macrophage mechanisms *STAT3*, *AHR*, and *CCR2* and neurodevelopmental and stemness mechanism *OLIG2* into three communities. Community 5 (C5) links *STAT3*, *AHR*, and *CCR2*, and C1 links *AHR* and *CCR2* as activators of shared modules. Modules regulated by *OLIG2* are represented in C2 with *OLIG2* as activator of its modules. Of note, C2 also contains a module that links *OLIG2* and *CCR2* co-acting as repressor and activator, respectively (Fig 6). Imaging-Community-AMARETTO also uncovered *THBS1* and *MAP2* as novel master drivers across the three *STAT3*, *AHR*, *CCR2*, and *OLIG2* communities that provide new insights into how these distinct key mechanisms are linked in glioma. *THBS1* is an activator driver of three modules in C1 and C5, and a repressor driver of three modules in C2. *MAP2* is a repressor driver of three modules in C1 and C5 and an activator driver of six modules in C2 (except repressor of TCGA GBM module 98). Taken together, C1 links *AHR* and *THBS1* (TCGA GBM module 79). C2 links *OLIG2, MAP2,* and *THBS1* (TCGA GBM module 75; Fig 5); *OLIG2* and *MAP2* (IvyGAP GBM module 38, TCGA GBM module 61); *CCR2*, *OLIG2*, and *MAP2* (TCGA GBM module 98; Fig 6). C5 links CCR2, *AHR*, and *THBS1* (IvyGAP GBM module 64; Fig 3) and *AHR* and *STAT3* (TCGA LGG module 125; Fig 2). Using genetic knockdown experiments of *THBS1* from LINCS/Connectivity Map, we confirmed that *THBS1* acts as activator and repressor drivers of its targets in IvyGAP GBM module 64 (Fig 3) and TCGA GBM module 75 (Fig 5), respectively (http://portals.broadinstitute.org/pochetlab/JCO_CCI_Imaging-AMARETTO/Imaging-AMARETTO_HTML_Report_TCGA-GBM_IVYGAP-GBM_TCGA-LGG/; www.ndexbio.org/#/network/16820740-d7ea-11e9-bb65-0ac135e8bacf).

Users are provided with all results in the form of hypertext markup language (HTML) reports that are generated in an automated manner for individual cohorts using Imaging-AMARETTO and multiple cohorts using Imaging-Community-AMARETTO. These reports include searchable tables within and across modules and communities, including statistics (ie, coefficients, *P* values, false discovery rate [FDR] values) for functional enrichment, driver validation, clinical and molecular biomarkers, and radiography and histopathology imaging features. These reports also include heat map visualizations for modules (Figs 2-6) and graph visualizations for communities (Appendix Fig A1). Source code is also provided to convert Imaging-AMARETTO and Imaging-Community-AMARETTO networks for depositing networks in the NDEx^55^ network database, taking advantage of its interactive features.

## RESULTS

To demonstrate its utility, we applied the Imaging-AMARETTO workflow to three studies of brain tumors: multiomics profiles from approximately 500 patients and approximately 30 radiography MRI features for approximately 200 patients from the TCGA GBM cohort,^56^ multiomics profiles from approximately 500 patients and approximately 30 radiography MRI features for approximately 180 patients from the TCGA LGG cohort,^56^ and for a subset of approximately 30 patients from the TCGA GBM patient cohort 270 transcriptomic profiles refined through histopathology imaging and annotated with imaging features that characterize anatomic structures for 122 samples and cancer stem cells for 148 samples were used from the IvyGAP GBM project.^57^

Disease progression in glioma is characterized by infiltration of resident microglia and peripheral macrophages in the tumor microenvironment and by pervasive infiltration of tumor cells in the healthy surroundings of the tumor.^58^ Understanding microglia and macrophage physiology and its complex interactions with tumor cells can elucidate their roles in glioma progression and uncover potentially interesting druggable targets.

Our results show that Imaging-AMARETTO captures these hallmarks of glioma, for example, key drivers of tumor-associated microglia and macrophage mechanisms^59^ mediated by *STAT3*, *AHR*, and *CCR2*, and neurodevelopmental and stemness mechanisms^60^ that involve *OLIG2*. Our findings recapitulate recent discoveries^59,60^ and provide interpretation of the molecular mechanisms in light of imaging biomarkers of clinical outcomes. Of note, Imaging-Community-AMARETTO also uncovers novel key master drivers that are shared by these distinct key mechanisms.

### Imaging-AMARETTO Deciphers Clinical Relevance of Multiomics Modules of Key Driver Mechanisms

Of clinical relevance, higher expression levels of *STAT3*, *AHR*, and *CCR2* modules (Figs 2, 3, and 6) are associated with shorter survival in GBM and LGG, and these modules also distinguish between molecular subclasses of GBM and LGG. In GBM, the mesenchymal subclass is represented by higher expression of these modules compared with the classical and proneural subclasses. In LGG, the astrocytoma subtype is characterized by higher expression of these modules compared with the oligodendroglioma subtype.

Diametrically opposed, higher expression levels of *OLIG2* modules (Figs 4-6) are associated with better survival in GBM and LGG, and these modules also distinguish between molecular subclasses of GBM and LGG but in the opposite direction. In GBM the classical and proneural subclasses are represented by higher expression of these modules compared with the mesenchymal subclass. In LGG, the oligodendroglioma subtype is characterized by higher expression of these modules compared with the astrocytoma subtype.

### Imaging-AMARETTO Deciphers Histopathology Imaging Biomarkers of Key Driver Mechanisms

Histopathology imaging features of anatomic structures show that higher expression of *STAT3*, *AHR*, and *CCR2* modules (Fig 3) distinguishes between samples derived from the cellular tumor compared with those from leading edge and infiltrating tumor regions. Higher expression of *OLIG2* modules distinguishes infiltrating tumor from cellular tumor samples.

Features representative of cancer stem cells show that higher expression of *STAT3*, *AHR*, and *CCR2* modules (Fig 3) distinguishes cancer stem-cell samples from their non–stem cancer cell counterparts. This observation is consistent across the distinct substructures of the cellular tumor, including hyperplastic blood vessels, microvascular proliferation, perinecrotic zone, and pseudopalisading cells around necrosis. Diametrically opposed, higher expression of *OLIG2* modules distinguishes non–stem cancer cells from cancer stem cells consistently across these microvascular and necrosis substructures.

### Imaging-AMARETTO Deciphers Radiography Imaging Biomarkers of Key Driver Mechanisms

Radiographic image features of *STAT3*, *AHR*, and *CCR2* modules (Figs 2 and 6) are highly consistent across GBM and LGG. Higher expression is associated with a higher proportion of enhancing tumor, lower proportion of nonenhancing tumor, and less cortical involvement. These modules also distinguish between measures of thickness of enhancing margin in both GBM and LGG. In GBM these *STAT3*, *AHR*, and *CCR2* modules show higher expression in association with eloquent cortex, while in LGG, they show higher expression in association with enhancement intensity.

Features of *OLIG2* modules (Figs 4-6) are also consistent across GBM and LGG and diametrically opposed to those of *STAT3*, *AHR*, and *CCR2*. In both GBM and LGG, higher expression is associated with a higher proportion of nonenhancing tumor and lower proportion of enhancing tumor. In GBM, higher expression is also associated within speech receptive eloquent cortex, while in LGG, higher expression is associated with cortical involvement and the presence of cysts.

### Imaging-Community-AMARETTO Uncovers Known Key and Novel Master Drivers Linking Mechanisms

Recent discoveries of *STAT3*, *AHR*, and *CCR2* as drivers of tumor-associated microglia and macrophage mechanisms^59^ are captured by modules in communities 1 and 5: TCGA LGG module 125 (Fig 2) shows hypomethylation of *STAT3* as activator driver of *AHR*, with higher expression associated with shorter survival and astrocytoma LGG, and IvyGAP GBM module 64 (Fig 3) shows that higher expression of *AHR* and *CCR2* is associated with the presence of cancer stem cells and microvascular substructures and suggests as novel activator driver, *THBS1*, that plays important roles in macrophage infiltration and angiogenesis,^61^ vascularization,^62^ and tumorigenesis^62^ in glioma. *OLIG2* as a driver of neurodevelopmental and stemness mechanisms^60,63,64^ is captured by modules in community 2: (1) TCGA LGG module 91 (Fig 4) shows hypomethylation of *OLIG2* as activator driver of this module, with higher expression associated with better survival, oligodendrocyte LGG, and *IDH1* wild-type status; (2) TCGA GBM module 75 (Fig 5) is driven by *OLIG2*, with higher expression associated with proneural and classical versus mesenchymal GBM, and suggests as novel repressor driver THBS1 and as novel activator driver hypomethylation of neuronal marker *MAP2* that plays important roles in microtubule-associated neurogenesis^65,66^ and reduces invasiveness^67^ and stemness^68^ in glioma; and (3) TCGA GBM module 98 (Fig 6) shows *CCR2* and *OLIG2* co-acting as activator and repressor drivers, respectively, highlighting their diametrically opposed behavior, with higher *CCR2* and lower *OLIG2* expression associated with mesenchymal versus proneural and classical GBM and suggesting as novel repressor driver hypomethylation of *MAP2*, consistent with observations in TCGA GBM module 75 (Fig 5). Using knockdown experiments of *THBS1* from LINCS/CMAP, we confirmed that *THBS1* acts as activator and repressor of its targets in IvyGAP GBM module 64 (Fig 3) and TCGA GBM module 75 (Fig 5), respectively.

Thus, Imaging-Community-AMARETTO (Appendix Fig A1) identified *THBS1* and *MAP2* as novel master drivers across the three *STAT3*, *AHR*, *CCR2*, and *OLIG2* communities that provide new insights into how these distinct key mechanisms are linked in glioma. Interesting avenues for further exploration with experimental validation studies include testing novel hypotheses of *THBS1* and *MAP2* as master regulators of shared mechanisms that involve macrophage infiltration, vascularization, tumorigenesis, invasion, stemness, and neurogenesis in glioma.

## DISCUSSION

We developed the Imaging-AMARETTO algorithms and software tools for imaging genomics to facilitate systematic interrogation of regulatory networks derived from multiomics data within and across related patient studies for their relevance to radiography and histopathology imaging features that predict clinical outcomes. We demonstrated its utility through application to three patient studies of brain tumors, including multiomics and radiography imaging data from the TCGA GBM and LGG studies and transcriptional and histopathology imaging data from the IvyGAP GBM study.

Our results show that Imaging-AMARETTO recapitulates known key drivers of tumor-associated microglia and macrophage mechanisms (*STAT3, AHR*, and *CCR2*) and neurodevelopmental and stemness mechanisms (*OLIG2*). Imaging-AMARETTO provides interpretation of the underlying molecular mechanisms in light of imaging biomarkers of clinical outcomes, and Imaging-Community-AMARETTO also uncovered novel master drivers *THBS1* and *MAP2* that establish relationships across these distinct mechanisms.

Of note, the querying of the Imaging-AMARETTO networks for modules whose elevated expression is inversely associated with proportions of enhancing tumor and cancer stem cells on radiography and histopathology imaging, respectively, shows that these modules are putatively coregulated by activator drivers *OLIG2* and *MAP2* and repressor drivers *STAT3*, *AHR*, *CCR2*, and *THBS1*. Thus, we hypothesize that restoration of the function of *OLIG2* and *MAP2* and attenuation of the expression of *STAT3*, *AHR*, *CCR2*, and *THBS1* potentially shift their target genes’ expression to more benign functional states associated with better survival in GBM and LGG.

This case study illustrates how Imaging-AMARETTO can be used for imaging diagnostics and prognostics by interrogating multimodal and multiscale networks for imaging biomarkers to identify their clinically relevant underlying molecular mechanisms. Our network-based imaging genomics tools are powerful hypothesis generators that facilitate the testing of known hypotheses and uncovering of novel hypotheses for follow-up with experimental validation studies. We anticipate that our tools for network-based fusion of multiomics, clinical, and imaging data will lead to better diagnostic and prognostic models of cancer and will open new avenues for drug discovery by integrating pharmacogenomic data into these networks, toward better therapeutics of cancer.^69,70^

## SOFTWARE AND RESOURCES

The source code of Imaging-AMARETTO is available from GitHub, and a Notebook for its application to the three brain cancer cohorts is available from Google Colaboratory, including links to interactive HTML reports and NDEx networks: http://portals.broadinstitute.org/pochetlab/JCO_CCI_Imaging-AMARETTO/Imaging-AMARETTO_Software_Resources.html.

## References

[1] National Cancer Institute: The Cancer Genome Atlas Program, 2018. https://www.cancer.gov/about-nci/organization/ccg/research/structural-genomics/tcga

[2] National Cancer Institute Office of Cancer Clinical Proteomics Research: OCCPR: A leader in cancer proteomics and proteogenomics, 2019. https://proteomics.cancer.gov

[3] The Cancer Imaging Archive: Welcome to The Cancer Imaging Archive, 2019. https://www.cancerimagingarchive.net

[4] Cancer Genome Atlas Research Network: Comprehensive genomic characterization defines human glioblastoma genes and core pathways. Nature 455:1061-1068, 2008 [Erratum: Nature 494:506, 2013] 10.1038/nature07385 PMC2671642 18772890

[5] Brennan CW, Verhaak RGW, McKenna A, et al: The somatic genomic landscape of glioblastoma. Cell 155:462-477, 2013 [Erratum: Cell 157:753, 2014] 10.1016/j.cell.2013.09.034 PMC3910500 24120142

[6] Brat DJ, Verhaak RGW, Aldape KD, et al: Comprehensive, integrative genomic analysis of diffuse lower-grade gliomas. N Engl J Med 372:2481-2498, 2015 10.1056/NEJMoa1402121 PMC4530011 26061751

[7] Bakas S, Akbari H, Sotiras A, et al: Advancing The Cancer Genome Atlas glioma MRI collections with expert segmentation labels and radiomic features. Sci Data 4:170117, 2017 10.1038/sdata.2017.117 PMC5685212 28872634

[8] Cancer Imaging Archive: VASARI Research Project. https://wiki.cancerimagingarchive.net/display/Public/VASARI+Research+Project

[9] Cancer Imaging Archive: TCGA Glioma Phenotype Research Group. https://wiki.cancerimagingarchive.net/display/Public/TCGA+Glioma+Phenotype+Research+Group

[10] Patel AP Tirosh I Trombetta JJ et al Single-cell RNA-seq highlights intratumoral heterogeneity in primary glioblastoma Science 344 1396 1401 2014 24925914 10.1126/science.1254257 PMC4123637

[11] Tirosh I, Venteicher AS, Hebert C, et al: Single-cell RNA-seq supports a developmental hierarchy in human oligodendroglioma. Nature 539:309-313, 2016 10.1038/nature20123 PMC5465819 27806376

[12] Filbin MG, Tirosh I, Hovestadt V, et al: Developmental and oncogenic programs in H3K27M gliomas dissected by single-cell RNA-seq. Science 360:331-335, 2018 10.1126/science.aao4750 PMC5949869 29674595

[13] Puchalski RB, Shah N, Miller J, et al: An anatomic transcriptional atlas of human glioblastoma. Science 360:660-663, 2018 10.1126/science.aaf2666 PMC6414061 29748285

[14] IvyGAP: Ivy Glioblastoma Atlas Project, 2019. https://glioblastoma.alleninstitute.org

[15] IvyGAP: Data download. https://glioblastoma.alleninstitute.org/static/download.html

[16] Rutman AM Kuo MD Radiogenomics: Creating a link between molecular diagnostics and diagnostic imaging Eur J Radiol 70 232 241 2009 19303233 10.1016/j.ejrad.2009.01.050

[17] Segal E Sirlin CB Ooi C et al Decoding global gene expression programs in liver cancer by noninvasive imaging Nat Biotechnol 25 675 680 2007 17515910 10.1038/nbt1306

[18] Diehn M Nardini C Wang DS et al Identification of noninvasive imaging surrogates for brain tumor gene-expression modules Proc Natl Acad Sci U S A 105 5213 5218 2008 18362333 10.1073/pnas.0801279105 PMC2278224

[19] Gevaert O Xu J Hoang CD et al Non-small cell lung cancer: Identifying prognostic imaging biomarkers by leveraging public gene expression microarray data--methods and preliminary results Radiology 264 387 396 2012 22723499 10.1148/radiol.12111607 PMC3401348

[20] Itakura H Achrol AS Mitchell LA et al Magnetic resonance image features identify glioblastoma phenotypic subtypes with distinct molecular pathway activities Sci Transl Med 7 303ra138 2015 10.1126/scitranslmed.aaa7582 PMC4666025 26333934

[21] Cheerla A Gevaert O Deep learning with multimodal representation for pancancer prognosis prediction Bioinformatics 35 i446 i454 2019 31510656 10.1093/bioinformatics/btz342 PMC6612862

[22] Momeni A, Thibault M, Gevaert O: Dropout-enabled ensemble learning for multi-scale biomedical data. in Crimi A, Bakas S, Kuijf H, et al (eds): Brainlesion: Glioma, Multiple Sclerosis, Stroke and Traumatic Brain Injuries. Cham, Switzerland, Springer International Publishing, 2019, pp 407-415

[23] Broad Institute: The *AMARETTO framework for network biology and medicine: Linking disease, drivers, targets and drugs via graph-based fusion of multi-omics, clinical, imaging, and perturbation data. http://portals.broadinstitute.org/pochetlab/amaretto.html

[24] GitHub: broadinstitute/ImagingAMARETTO. https://github.com/broadinstitute/ImagingAMARETTO

[25] Google Colaboratory: Imaging-AMARETTO: An imaging genomics software tool to systematically interrogate multi-omics networks for relevance to radiography and histopathology imaging biomarkers of clinical outcomes with application to studies of brain tumors. https://colab.research.google.com/drive/14u1KZJ3Gf-9qjDycyBKzBiN5VzzOa2xU#scrollTo=LujO14znmO0J

[26] Stubbs BJ, Gopaulakrishnan S, Glass K, et al: TFutils: Data structures for transcription factor bioinformatics. F1000Res 8:152, 2019 10.12688/f1000research.17976.1 PMC6600865 31297189

[27] Carey V, Gopaulakrishnan S: TFutils: Bioconductor version release (3.10). https://bioconductor.org/packages/TFutils

[28] Subramanian A Tamayo P Mootha VK et al Gene set enrichment analysis: A knowledge-based approach for interpreting genome-wide expression profiles Proc Natl Acad Sci U S A 102 15545 15550 2005 16199517 10.1073/pnas.0506580102 PMC1239896

[29] Liberzon A Birger C Thorvaldsdóttir H et al The Molecular Signatures Database (MSigDB) Hallmark gene set collection Cell Syst 1 417 425 2015 26771021 10.1016/j.cels.2015.12.004 PMC4707969

[30] COSMIC: COSMIC, the Catalogue of Somatic Mutations in Cancer. https://cancer.sanger.ac.uk/cosmic 10.1093/nar/gky1015 PMC6323903 30371878

[31] Mermel CH Schumacher SE Hill B et al GISTIC2.0 facilitates sensitive and confident localization of the targets of focal somatic copy-number alteration in human cancers Genome Biol 12 R41 2011 21527027 10.1186/gb-2011-12-4-r41 PMC3218867

[32] Cedoz PL, Prunello M, Brennan K, et al: MethylMix 2.0: An R package for identifying DNA methylation genes. Bioinformatics 34:3044-3046, 2018 10.1093/bioinformatics/bty156 PMC6129298 29668835

[33] Gevaert O Tibshirani R Plevritis SK Pancancer analysis of DNA methylation-driven genes using MethylMix Genome Biol 16 17 2015 25631659 10.1186/s13059-014-0579-8 PMC4365533

[34] Champion M Brennan K Croonenborghs T et al Module analysis captures pancancer genetically and epigenetically deregulated cancer driver genes for smoking and antiviral response EBioMedicine 27 156 166 2018 29331675 10.1016/j.ebiom.2017.11.028 PMC5828545

[35] Shinde J, Everaert C, Bakr S, et al: AMARETTO: Regulatory network inference and driver gene evaluation using integrative multi-omics analysis and penalized regression: Bioconductor version release 3.10, 2019. https://bioconductor.org/packages/AMARETTO

[36] Gevaert O, Villalobos V, Sikic BI, et al: Identification of ovarian cancer driver genes by using module network integration of multi-omics data. Interface Focus 3:20130013, 2013 [Erratum Interface Focus 4:20140023, 2014] 10.1098/rsfs.2013.0013 PMC3915833 24511378

[37] Gevaert O Plevritis S Identifying master regulators of cancer and their downstream targets by integrating genomic and epigenomic features Pac Symp Biocomput 123 134 2013 23424118 PMC3911770

[38] Zou H Hastie T Regularization and variable selection via the elastic net J R Stat Soc Series B Stat Methodol 67 301 320 2005

[39] Newman AM Liu CL Green MR et al Robust enumeration of cell subsets from tissue expression profiles Nat Methods 12 453 457 2015 25822800 10.1038/nmeth.3337 PMC4739640

[40] Stanford University: CIBERSORT. https://cibersort.stanford.edu

[41] Ben-Porath I Thomson MW Carey VJ et al An embryonic stem cell-like gene expression signature in poorly differentiated aggressive human tumors Nat Genet 40 499 507 2008 18443585 10.1038/ng.127 PMC2912221

[42] Marson A Levine SS Cole MF et al Connecting microRNA genes to the core transcriptional regulatory circuitry of embryonic stem cells Cell 134 521 533 2008 18692474 10.1016/j.cell.2008.07.020 PMC2586071

[43] Kim J Woo AJ Chu J et al A Myc network accounts for similarities between embryonic stem and cancer cell transcription programs Cell 143 313 324 2010 20946988 10.1016/j.cell.2010.09.010 PMC3018841

[44] Newman MEJ Girvan M Finding and evaluating community structure in networks Phys Rev E Stat Nonlin Soft Matter Phys 69 026113 2004 14995526 10.1103/PhysRevE.69.026113

[45] Baryawno N Przybylski D Kowalczyk MS et al A cellular taxonomy of the bone marrow stroma in homeostasis and leukemia Cell 177 1915 1932.e16 2019 31130381 10.1016/j.cell.2019.04.040 PMC6570562

[46] ENCODE Project Consortium The ENCODE (ENCyclopedia Of DNA Elements) Project Science 306 636 640 2004 15499007 10.1126/science.1105136

[47] Lachmann A Xu H Krishnan J et al ChEA: Transcription factor regulation inferred from integrating genome-wide ChIP-X experiments Bioinformatics 26 2438 2444 2010 20709693 10.1093/bioinformatics/btq466 PMC2944209

[48] Rouillard AD, Gundersen GW, Fernandez NF, et al: The Harmonizome: A collection of processed datasets gathered to serve and mine knowledge about genes and proteins. Database (Oxford) 2016:baw100, 2016 10.1093/database/baw100 PMC4930834 27374120

[49] Lamb J Crawford ED Peck D et al The Connectivity Map: Using gene-expression signatures to connect small molecules, genes, and disease Science 313 1929 1935 2006 17008526 10.1126/science.1132939

[50] Subramanian A Narayan R Corsello SM et al A next generation Connectivity Map: L1000 platform and the first 1,000,000 profiles Cell 171 1437 1452.e17 2017 29195078 10.1016/j.cell.2017.10.049 PMC5990023

[51] Verhaak RGW Hoadley KA Purdom E et al Integrated genomic analysis identifies clinically relevant subtypes of glioblastoma characterized by abnormalities in PDGFRA, IDH1, EGFR, and NF1 Cancer Cell 17 98 110 2010 20129251 10.1016/j.ccr.2009.12.020 PMC2818769

[52] Gevaert O Mitchell LA Achrol AS et al Glioblastoma multiforme: Exploratory radiogenomic analysis by using quantitative image features Radiology 273 168 174 2014 24827998 10.1148/radiol.14131731 PMC4263772

[53] Liu TT Achrol AS Mitchell LA et al Magnetic resonance perfusion image features uncover an angiogenic subgroup of glioblastoma patients with poor survival and better response to antiangiogenic treatment Neuro-oncol 19 997 1007 2017 28007759 10.1093/neuonc/now270 PMC5570189

[54] Nicolasjilwan M Hu Y Yan C et al Addition of MR imaging features and genetic biomarkers strengthens glioblastoma survival prediction in TCGA patients J Neuroradiol 42 212 221 2015 24997477 10.1016/j.neurad.2014.02.006 PMC5511631

[55] NDEx: Welcome to the Network Data Exchange. https://home.ndexbio.org

[56] GenePattern: AMARETTO supporting data files. https://datasets.genepattern.org/?prefix=data/module_support_files/Amaretto

[57] Carey V: ivygapSE: A SummarizedExperiment for Ivy-GAP data: Bioconductor version release 3.10, 2019. https://bioconductor.org/packages/ivygapSE

[58] Sevenich L Brain-resident microglia and blood-borne macrophages orchestrate central nervous system inflammation in neurodegenerative disorders and brain cancer Front Immunol 9 697 2018 29681904 10.3389/fimmu.2018.00697 PMC5897444

[59] Takenaka MC, Gabriely G, Rothhammer V, et al: Control of tumor-associated macrophages and T cells in glioblastoma via AHR and CD39. Nat Neurosci 22:729-740, 2019 [Erratum: Nat Neurosci 22:1533, 2019] 10.1038/s41593-019-0370-y PMC8052632 30962630

[60] Krichevsky AM Uhlmann EJ Oligonucleotide therapeutics as a new class of drugs for malignant brain tumors: Targeting mRNAs, regulatory RNAs, mutations, combinations, and beyond Neurotherapeutics 16 319 347 2019 30644073 10.1007/s13311-018-00702-3 PMC6554258

[61] Offer S Menard JA Pérez JE et al Extracellular lipid loading augments hypoxic paracrine signaling and promotes glioma angiogenesis and macrophage infiltration J Exp Clin Cancer Res 38 241 2019 31174567 10.1186/s13046-019-1228-6 PMC6556032

[62] Daubon T, Léon C, Clarke K, et al: Deciphering the complex role of thrombospondin-1 in glioblastoma development. Nat Commun 10:1146, 2019 10.1038/s41467-019-08480-y PMC6408502 30850588

[63] Suvà ML Rheinbay E Gillespie SM et al Reconstructing and reprogramming the tumor-propagating potential of glioblastoma stem-like cells Cell 157 580 594 2014 24726434 10.1016/j.cell.2014.02.030 PMC4004670

[64] Ceccarelli M Barthel FP Malta TM et al Molecular profiling reveals biologically discrete subsets and pathways of progression in diffuse glioma Cell 164 550 563 2016 26824661 10.1016/j.cell.2015.12.028 PMC4754110

[65] Gao L, Huang S, Zhang H, et al: Suppression of glioblastoma by a drug cocktail reprogramming tumor cells into neuronal like cells. Sci Rep 9:3462, 2019 [Erratum: Sci Rep 10:2971, 2020] 10.1038/s41598-019-39852-5 PMC6401026 30837577

[66] Yuan J Zhang F Hallahan D et al Reprogramming glioblastoma multiforme cells into neurons by protein kinase inhibitors J Exp Clin Cancer Res 37 181 2018 30071868 10.1186/s13046-018-0857-5 PMC6090992

[67] Zhou Y Wu S Liang C et al Transcriptional upregulation of microtubule-associated protein 2 is involved in the protein kinase A-induced decrease in the invasiveness of glioma cells Neuro-oncol 17 1578 1588 2015 26014048 10.1093/neuonc/nov060 PMC4633926

[68] Yi R Feng J Yang S et al miR-484/MAP2/c-Myc-positive regulatory loop in glioma promotes tumor-initiating properties through ERK1/2 signaling J Mol Histol 49 209 218 2018 29480405 10.1007/s10735-018-9760-9

[69] Emmert-Streib F Dehmer M Haibe-Kains B Gene regulatory networks and their applications: Understanding biological and medical problems in terms of networks Front Cell Dev Biol 2 38 2014 25364745 10.3389/fcell.2014.00038 PMC4207011

[70] Wooden B Goossens N Hoshida Y et al Using big data to discover diagnostics and therapeutics for gastrointestinal and liver diseases Gastroenterology 152 53 67.e3 2017 27773806 10.1053/j.gastro.2016.09.065 PMC5193106

[71] Imaging-AMARETTO HTML report of module 125 from the TCGA LGG cohort. http://portals.broadinstitute.org/pochetlab/JCO_CCI_Imaging-AMARETTO/Imaging-AMARETTO_HTML_Report_TCGA-GBM_IVYGAP-GBM_TCGA-LGG/TCGA_LGG/AMARETTOhtmls/modules/module125.html

[72] Imaging-AMARETTO HTML report of module 64 from the IvyGAP GBM cohort. http://portals.broadinstitute.org/pochetlab/JCO_CCI_Imaging-AMARETTO/Imaging-AMARETTO_HTML_Report_TCGA-GBM_IVYGAP-GBM_TCGA-LGG/Ivygap_GBM/AMARETTOhtmls/modules/module64.html

[73] Imaging-AMARETTO HTML report of module 91 from the TCGA LGG cohort. http://portals.broadinstitute.org/pochetlab/JCO_CCI_Imaging-AMARETTO/Imaging-AMARETTO_HTML_Report_TCGA-GBM_IVYGAP-GBM_TCGA-LGG/TCGA_LGG/AMARETTOhtmls/modules/module91.html

[74] Imaging-AMARETTO HTML report of module 75 from the TCGA GBM cohort. http://portals.broadinstitute.org/pochetlab/JCO_CCI_Imaging-AMARETTO/Imaging-AMARETTO_HTML_Report_TCGA-GBM_IVYGAP-GBM_TCGA-LGG/TCGA_GBM/AMARETTOhtmls/modules/module75.html

[75] Imaging-AMARETTO HTML report of module 98 from the TCGA GBM cohort. http://portals.broadinstitute.org/pochetlab/JCO_CCI_Imaging-AMARETTO/Imaging-AMARETTO_HTML_Report_TCGA-GBM_IVYGAP-GBM_TCGA-LGG/TCGA_GBM/AMARETTOhtmls/modules/module98.html

